# Dynamic Regulation of Cysteine Oxidation and Phosphorylation in Myocardial Ischemia–Reperfusion Injury

**DOI:** 10.3390/cells10092388

**Published:** 2021-09-11

**Authors:** Kevin M. Casin, John W. Calvert

**Affiliations:** Carlyle Fraser Heart Center, Department of Surgery, Division of Cardiothoracic Surgery, Emory University School of Medicine, Atlanta, GA 30322, USA; kcasin@emory.edu

**Keywords:** heart, ischemia, reperfusion, oxidative stress, reactive oxygen species, redox signaling, AMPK, Akt, PKA, PKG

## Abstract

Myocardial ischemia–reperfusion (I/R) injury significantly alters heart function following infarct and increases the risk of heart failure. Many studies have sought to preserve irreplaceable myocardium, termed cardioprotection, but few, if any, treatments have yielded a substantial reduction in clinical I/R injury. More research is needed to fully understand the molecular pathways that govern cardioprotection. Redox mechanisms, specifically cysteine oxidations, are acute and key regulators of molecular signaling cascades mediated by kinases. Here, we review the role of reactive oxygen species in modifying cysteine residues and how these modifications affect kinase function to impact cardioprotection. This exciting area of research may provide novel insight into mechanisms and likely lead to new treatments for I/R injury.

## 1. Introduction

Myocardial ischemia–reperfusion (I/R) injury often precedes more severe forms of cardiovascular disease (i.e., heart failure) [1]. Ischemia is induced by either a thrombotic or spastic occlusion of the coronary arteries, which feed the heart, and leads to a series of pathological changes that culminate in the death of cardiomyocytes [2]. Subsequent reperfusion—the restoration of blood flow—exacerbates cell death by introducing reactive oxygen species (ROS), which damage macromolecules, such as DNA, proteins, and lipids [3]. Each insult further impedes heart function, ultimately resulting in heart failure and patient death.

While studies have strived to reduce I/R injury, clinical treatments have failed to drastically reduce cell death and preserve myocardium. Yet, significant progress has been made to understand the molecular underpinnings of cardioprotection in preclinical models and some hope remains to effectively reduce I/R injury. Kinases—enzymes that extract phosphate groups from adenosine triphosphate (ATP) and catalyze protein phosphorylation—are integral components of cardioprotective pathways, and modification of their activity is a promising area of research. Reduction–oxidation (redox) reactions are crucial processes that help maintain cardiac homeostasis and are known to regulate cell signaling pathways. Recent work has shown how kinases are often products of redox reactions, which modify phosphorylation events [4]. However, the impact of these modifications on cardioprotection and I/R injury remains to be fully understood.

This review aims to summarize important, recent work studying the cysteine oxidation of kinases essential to cardioprotection and highlight an emerging area of research. Importantly, methionine oxidation can also regulate protein kinases (e.g., calcium-calmodulin kinase II); however, this modification is beyond the scope of this review (please see Moskovitz and Smith. (2021)) [5,6]. Novel studies are needed to further understand the molecular processes that govern cardiac homeostasis and protection. Harnessing and exploiting these pathways may provide a road for new therapeutics.

## 2. Myocardial Ischemia–Reperfusion Injury

### 2.1. Pathophysiology of Ischemia

Ischemia is induced by the restriction of blood flow to tissues, and diminished oxygen supply. While hypoxia stimulates several cell survival responses, prolonged oxygen limitation can initiate cell death pathways [7]. Myocardial ischemia is characterized by periods of ATP depletion, pH changes, and calcium dysregulation [3].

Oxygen serves as the final electron acceptor in the electron transport chain (ETC) which drives the mitochondrial ATP synthases to yield the ATP necessary for cellular function. Restricting oxygen flow impairs the ETC and reduces ATP output. As a result of a homeostatic need to maintain ATP production, cellular metabolism relies on glucose and other non-oxidative processes for energy [8]. The sodium–potassium pump, which is responsible for maintaining ionic balance within a cell by exporting sodium and importing potassium in a 3:2 ratio, is an ATP-dependent protein [9]. Along with the sodium–hydrogen pump that maintains intracellular sodium concentrations by expelling hydrogen for sodium, ischemia leads to the dysfunction of both proteins and severely impairs the delicate ion balance of the cell [10,11]. Without energy, the sodium–potassium pump stops, leading to a build-up of sodium, and the sodium–hydrogen pump—which does not rely on ATP—changes direction, expelling excess sodium in exchange for hydrogen [12]. Along with a byproduct of substrate-level phosphorylation, or glycolysis-dependent metabolism, which is lactic acid, intracellular pH falls and damages organelles [13,14,15].

Calcium is another essential ion and a key component of cardiomyocyte function by serving as the regulator of muscle contraction. As comprehensibly reviewed by Bers (2002), and here briefly, excitation–contraction coupling begins with electrical stimulation—originating from the spontaneous depolarization of pacemaker cells (i.e., “funny” currents) [16,17]. This opens the L-type calcium channel and triggers the ryanodine receptor to release calcium from the sarcoplasmic reticulum (SR) [18,19]. Calcium then binds to cardiac troponin, which moves tropomyosin and allows for actin–myosin interactions—a key step for muscle contraction [20]. During relaxation, calcium is sequestered into the SR by the SR calcium transport ATPase (SERCA), regulated by phospholamban, and removed from the cell by the sodium–calcium exchanger [17]. During ischemia, excess sodium compels the sodium–calcium exchanger to import calcium in exchange for sodium removal [12]. Furthermore, the ATP dearth inhibits SERCA, causing a calcium buildup, and prevents the release of actin by myosin, maintaining muscle contraction and damaging cardiomyocytes [21]. Finally, calcium build-up activates calcium-dependent proteases, or calpains, which digest proteins and impair homeostasis [22,23]. Taken together, ischemia initiates a cell death cascade all linked to the obstruction of oxygen flow. Current interventions focus on the timely restoration of blood flow to the ischemic zone.

### 2.2. Pathophysiology of Reperfusion

Restoring blood flow in a timely manner is the only effective treatment for ischemia [24,25]. Refusion can be facilitated by blood thinners [26,27]. Mechanical reperfusion can also be performed by percutaneous coronary intervention, which uses a stent to widen a coronary artery narrowed by plaque, blood clot, or spasm [28,29]. Alternatively, ischemic myocardium can be reperfused with coronary bypass grafts by essentially forming a bridge over the obstruction [2,30]. While reperfusion is essential for treating ischemia, this procedure can cause injury to the heart by inducing cell death, and the heart progress into failure with time.

While the restriction of oxygen leads to severe damage, reperfusion exacerbates injury, partly by introducing an oxidative bolus [31]. This leads to the formation of various oxidative, nitrosative, and lipid radicals that lead to protein degradation and compromise cellular integrity [32,33]. Upon reperfusion, cytosolic calcium levels, already elevated by ischemia, increase further and activate calpains, which initiate apoptosis [34,35,36]. Along with the sudden correction in pH, these factors all converse at the mitochondria and lead to the opening of the mitochondrial permeability transition pore [37]. While the exact structure of the pore is still under active investigation, it is well understood that the opening of this pore is a key factor in the severity of I/R injury [38]. Opening of the pore depolarizes the mitochondrial membrane and initiates cell death [39]. Kinases can mitigate pore opening and reduce the detrimental effects of oxidative stress by activating cell survival pathways [40,41]. These studies implicate a crucial role for kinases in I/R injury and cardioprotection.

## 3. Kinases in Cardioprotection

### 3.1. Cardioprotection

The goal of cardioprotection is to reduce the severity of I/R injury and preserve myocardium by reducing cardiomyocyte death. In 1986, Murry et al., following the work of Reimer et al., showed that brief periods of ischemia (i.e., ischemic preconditioning) before I/R reduced injury [42,43]. Subsequent studies have discovered alternative conditioning strategies, such as ischemic postconditioning and remote preconditioning, and interrogated the molecular pathways governing these methods [44,45,46,47]. Brief periods of ischemia produce enough stress to trigger cell survival pathways that limit oxidative stress, inflammation, and apoptosis [48,49,50,51]. These mechanisms largely involve signal transduction from critical kinases, which integrate the ischemic stimulus with downstream responses [52]. In this review, we provide a brief overview of the major kinases involved in cardioprotection; however, this is not a comprehensive review, and we refer the reader to [53].

### 3.2. Regulation of Oxidant Proteins by Kinases

Enzymes responsible for generating oxidants, such as nitric oxide and ROS, are regulated by kinases, thus contributing to oxidative cysteine regulation. Constitutive isoforms of nitric oxide synthase (NOS), such as endothelial NOS (eNOS), release nitric oxide by metabolizing L-arginine in the presence of oxygen, nicotinamide adenine dinucleotide phosphate (NADPH), and calcium–calmodulin [54]. Nitric oxide leads to the stimulation of sGC and consequently activates PKG [55]. Furthermore, nitric oxide reversibly modifies cysteine residues on protein in an oxidative process known as S-nitrosation, which is beyond the scope of this review (please see Stromberski et al. 2019) [56]. The production of nitric oxide is regulated by the phosphorylation of NOS. Ser1177 is perhaps the most well-known phosphorylation site on eNOS because it is essential for enzyme activation [57,58]. This site is phosphorylated by AMPK, PKA, PKG, Akt, and others, showing that this residue is critical for cardioprotection [57,59,60,61]. Phosphorylation of Ser633 by PKA also activates eNOS [61]. Apart from activation, Thr495 inhibits eNOS by protein kinase C and likely AMPK, though this has only been shown in vitro [59,62]. eNOS regulation is a critical component of cardioprotection and an important target of protective kinases.

NADPH oxidase (NOX) enzymes reduce oxygen to form superoxide and are subsequently involved in signaling and immune pathways [63]. Five NOX isoforms are known, along with two dual oxidases [64]. NOX2 and NOX4 are abundantly expressed in cardiomyocytes and are also found in other cell types within the heart [63]. In 2013, Matsushima et al. showed that systemic NOX2 and NOX4 ablation increased infarct size following I/R injury [65,66]. These data suggest an important role for low-level ROS in the heart and, possibly, the physiological cardioprotective mechanism. Akt phosphorylation is reported to regulate a critical regulatory subunit, p47phox [67]. In turn, p47phox leads to the modulation of Akt signaling likely through the activation of NOX enzymes [68,69]. Taken together, kinases are critical in the regulation of various oxidative enzymes; NOX and NOS proteins are examples. While these enzymes are modulated by kinases, they can also control the activation or inhibition of the very kinases that regulate them, in turn.

### 3.3. PI3K/Akt in Myocardial Ischemia–Reperfusion Injury

**Figure 1:**
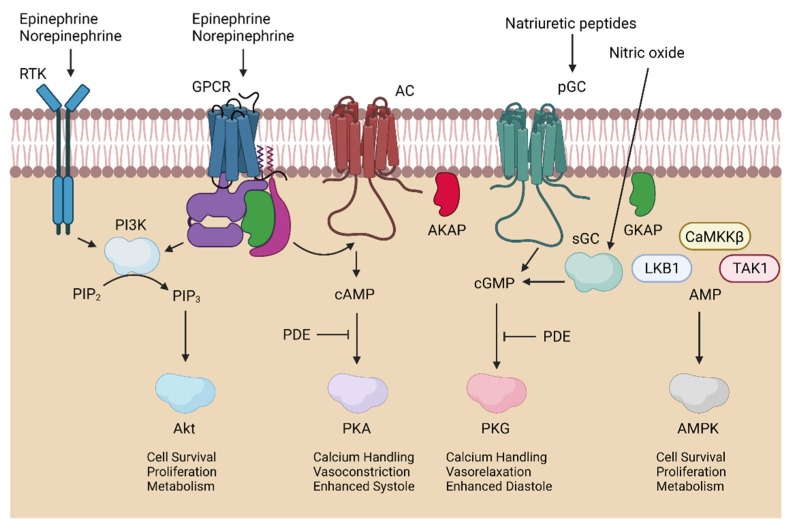
Summary of Cardioprotective Kinase Signaling Pathways. From left to right, receptor tyrosine kinases (RTK) or G-protein coupled receptors (GPCR) stimulate phosphoinositide 3-kinase (PI3K) to produce phosphatidylinositol-(3,4,5) triphosphate (PIP_3_) from phosphatidylinositol-(4,5) bisphosphate (PIP_2_) and leads to protein kinase B (Akt) activation. Protein kinase A (PKA) activity is induced by GPCR-mediated adenylyl cyclase (AC) stimulation, which produced cyclic adenosine monophosphate (cAMP), regulated by A-kinase-anchoring proteins (AKAP) and phosphodiesterases (PDE). Natriuretic peptides and nitric oxide, respectively, trigger particular and soluble guanylyl cyclase (pGC/sGC) to make cyclic guanosine monophosphate (cGMP), also regulated by PDE and G-kinase-anchoring proteins (GKAP), and initiate protein kinase G (PKG) activity. Finally, AMP kinase (AMPK) is activated by AMP, along with the following kinases: liver kinase B1 (LKB1), calmodulin-dependent protein kinase kinase β(CaMKKβ), and transformation growth factor-β-activated kinase-1 (TAK1).

An important cardioprotection pathway has been called the reperfusion injury salvage kinase (RISK) pathway and involves the enzymes phosphoinositide 3-kinase (PI3K) and protein kinase B (Akt) [52]. As depicted in Figure 1, membrane-bound receptor tyrosine kinases (e.g., Src) or G-protein coupled receptors stimulate PI3K to phosphorylate phosphatidylinositol-(4,5) bisphosphate (PIP2) or to phosphatidylinositol-(3,4,5) triphosphate (PIP3). This allows for Akt localization at the plasma membrane [70]. Akt may then be activated by mammalian target of rapamycin complex (mTORC) 2 and phosphoinositide-dependent kinase 1 [71]. Part of Akt signaling inhibits glycogen synthase kinase (GSK) 3β which, in turn, allows the modulation of cell survival, proliferation, and metabolism pathways [72]. Many studies have reported the cardioprotective effect of Akt.

Currently, three Akt isoforms are known. Knock-out mice demonstrate the importance of each isoform: Akt1 promotes cell survival, Akt2 deletion impairs glucose metabolism, and Akt3 supports brain development [73]. Akt1 and 2 are largely expressed in the heart, while Akt3 is predominately expressed in embryonic hearts [74,75]. All isoforms contained conserved Thr308 and Ser473 which are critical for transducing the Akt signaling pathway [76]. Activation of Akt by phosphorylation is an important component of cardioprotection [77]. Expression of a constitutively active Akt significantly reduces myocardial cell death and infarct size in rat hearts [78]. Furthermore, ischemic preconditioning activates Akt and phosphorylates GSK3β [79,80,81]. Genetic polymorphisms for Akt are reported to increase the risk of developing metabolic syndrome, which is a risk factor for cardiovascular disease [82,83,84]. Future studies are needed to understand the role of these polymorphisms on Akt function in I/R injury. Other kinases are also essential components of cardioprotection from I/R injury.

### 3.4. AMPK in Myocardial Ischemia–Reperfusion Injury

Adenosine monophosphate (AMP)-activated protein kinase (AMPK) is also a cardioprotective kinase intimately involved in the regulation of cellular metabolism (Figure 1). AMPK exists as a heterotrimer made from two α catalytic subunits (α1/α2), two β scaffolding subunits (β1/β2), and three γ regulatory subunits (γ1–3) [85,86]. AMPK is classically regulated by phosphorylation of Thr172, which acts as a biomarker for AMPK activation. Three kinases are known to phosphorylate AMPK: liver kinase B1 (LKB1), calmodulin-dependent protein kinase kinase (CaMKKβ), and transforming growth factor-β-activated kinase-1 (TAK1) [87,88]. AMPK activity can also be modulated by AMP binding to cystathionine β-synthase domain repeats in the γ1 subunit [85].

AMPK activation results in the stimulation of cellular metabolism and the production of ATP. AMPK upregulates glucose transport type 4, which increases glucose uptake, inhibits acetyl-CoA carboxylase 2 to activate fatty acid oxidation, and stimulates glycolysis by phosphorylating 6-phosphofructo-2-kinase isoforms specific to cardiomyocytes [86]. In the heart, AMPKα1 is predominantly expressed in fibroblasts, while AMPKα2 is mostly found in cardiomyocytes [89]. Additionally, both LKB1 and TAK1 are highly expressed in the heart [88]. However, while CaMKKβ has low cardiac expression, the function of calmodulin and its calcium-dependent regulation intimately links this kinase with contractile activity [86]. Cellular metabolism is an essential mechanism for cardioprotection and AMPK is an important hub for restoring ATP production after I/R injury.

Several studies have demonstrated that activation of AMPK protects hearts from I/R injury by reducing infarct size and maintaining cardiac function [90,91,92]. LKB1 cardiomyocyte-specific deletion induces cardiac hypertrophy and death within 6 months of age by preventing AMPKβ2 activation [93,94,95]. A dominant negative mutation of AMPKβ2 worsened post-I/R heart function and significantly increased cell death [96]. Furthermore, these hearts had dysregulated post-I/R fatty acid oxidation and glucose uptake. AMPKβ2 impairment also affected AMPKβ1 activity [96]. Loss of AMPKβ1 function has additional implications. Studies have demonstrated that AMPKβ1 deletion in myofibroblasts increases fibroblast proliferation post-I/R, exacerbated adverse left ventricular remodeling, and decreased expression of connexin 43—a critical gap junction protein in cardiomyocytes [89,97]. While AMPK is a critical mediator of cardioprotection, other kinases are also known to be important.

### 3.5. PKA in Myocardial Ischemia–Reperfusion Injury

Cyclic AMP (cAMP)-dependent protein kinase (PKA) is also a mediator of cardioprotection (Figure 1) [98]. PKA is activated by G-protein coupled receptors (e.g., β-adrenergic receptors) that stimulate adenylyl cyclase (AC), which produces the second messengers cAMP [99]. A-kinase-anchoring proteins (AKAPs) and phosphodiesterases (PDEs) compartmentalize cAMP and fine-tune the cAMP-mediated signaling cascade (as reviewed by Fischmeister et al. (2006)) [100]. For instance, isoproterenol- and glucagon-like peptide-1 elicit cAMP production but activate unique pathways due to cAMP localization [101,102]. PKA exists as a heterotetramer with two regulatory domains—where AKAPs and cAMP bind—and two catalytic domains, which phosphorylate serine and threonine residues on substrate proteins [99]. Many of these PKA substrates are regulators of heart contraction.

PKA is known to phosphorylate the L-type calcium channel, the ryanodine receptor, and phospholamban, along with troponin I and myosin binding protein-C. Altogether, phosphorylation increases intracellular calcium and drives muscle contraction. Thorough reviews of PKA’s role in I/R injury were previously published (please see reviews by Colombe and Pidoux (2021), and Liu et al., (2021)), but we will present a few key studies here [98,99]. Previous reports have shown that β2-adrenergic stimulation protects the heart from acute I/R injury by stimulating cAMP and activating the PKA-Akt axis [103]. The cardioprotective effects of β-adrenergic signaling largely depend on cAMP availability and dictate the role of PKA. Ordinary activation by cAMP leads to the inhibition of cytochrome c oxidase and reduces oxidative stress [104]. However, loss of the regulatory PKA subunit, as occurs with oxidation, exacerbates I/R injury and impairs the antioxidant transcriptional pathway [105,106]. These studies show the importance of PKA signaling in protecting from I/R injury.

### 3.6. PKG in Myocardial Ischemia–Reperfusion Injury

Cyclic guanosine monophosphate (cGMP)-dependent protein kinase (PKG) is another regulator of cardiac function involved in cardioprotection (Figure 1). Like cAMP, cGMP is produced by particulate and soluble guanylyl cyclase (GC) [107]. Natriuretic peptides bind to the extracellular portion of particulate GC (pGC), while nitric oxide stimulates soluble GC (sGC). Both generate cGMP and regulate PKG activity [108]. cGMP is also compartmentalized by G-kinase-anchoring proteins (GKAPs) [109]. PDE proteins help with localizing cGMP through cGMP hydrolysis, thus regulating PKG activity [110]. PKG has two isoforms: PKGI and PKGII. PKGIα is mainly expressed in the heart and is more sensitive to cGMP than PKG1β. PKG, along with PKA, also controls heart contraction [107,111].

PKG phosphorylates the L-type calcium channel, phospholamban, myosin-binding protein C, and troponin I; however, unlike PKA, PKG enhances vasodilation and heart relaxation (or diastole) [111]. Cardioprotection by PKG is likely best demonstrated by the potent reduction in infarct size in PDE5 inhibitors, such as sildenafil, along with activators of sGC [112,113,114]. These pharmaceuticals allow for cGMP levels to increase and stimulate PKG activity. Recently, Ranek et al. demonstrated that PDE5 overexpression exacerbated I/R injury by increasing the toxic accumulation of ubiquitinated proteins, both in neonatal rat ventricular myocytes and in vivo [115]. Other forms of elevating cGMP have also been shown to protect the heart. Activation of sGC either by pharmaceuticals or nitric oxide increases the activity of PKG and can lead to preserved mitochondrial integrity or reduced inflammation [112,116,117,118]. Like many other kinases, PKG can activate and regulate signaling pathways that can maintain cardiac homeostasis following I/R injury. However, these kinases can also regulate enzymes that increase oxidation.

## 4. Oxidation of Kinases and Pathological Consequences

### 4.1. Brief Overview of Redox Biology

Redox reactions are fundamentally structured around the movement of electrons. When a molecule gains an electron, it is considered a reduction reaction; conversely, when an electron is gained, the molecule is oxidized. This chemical balance is maintained within cells by both oxidants and antioxidants (or reductants) produced from biochemical reactions. Oxidants, such as ROS, are not only produced as metabolic byproducts, but also from enzymes [119]. Though reductants are beyond the scope of this review (but reviewed by Xiao and Loscalzo (2020)), antioxidant molecules (i.e., glutathione); enzymes (i.e., glutathione reductase); and proteins, such as thioredoxin (Trx), are also essential to maintaining redox homeostasis [120]. Together, this delicate balance sustains cardiomyocyte health and can drive pathology.

ROS are well-studied oxidants that involve the reduction in oxygen to yield species, such as singlet oxygen, superoxide, hydrogen peroxide (H_2_O_2_), and hydroxyl radicals [121]. The mitochondria is an important source of intracellular superoxide production that must be kept in check. Excessive ROS can damage macromolecules, such as DNA, proteins, and lipids, and ultimately lead to the release of cytochrome c to initiate apoptosis [122,123]. Superoxide is converted into H_2_O_2_ by superoxide dismutase [124]. Although these radicals are tightly regulated, they are essential participates in key signaling processes needed for homeostasis [125]. Conversely, an overload of reductants is also detrimental to cells [120]. While superoxide dismutase regulates mitochondrial ROS and keep radical levels in check by converting superoxide into hydrogen H_2_O_2_, catalase further processed H_2_O_2_ into water and oxygen [126,127]. Redox biology involves a delicate balance between oxidants and reductants to maintain homeostasis. These molecules participate in important signaling roles and can tune kinase pathways.

### 4.2. Kinase Oxidation in Myocardial Ischemia–Reperfusion Injury

Kinases are well-studied mediators of signal transduction, integrating most forms of cellular stimulants into a finely honed response. Seminal work from Sundaresan et al. (1995) and Bae et al. (1997) demonstrated that platelet-derived growth factor signaling is mediated, at least in part, by H_2_O_2_, showing that kinases are involved in redox-mediated signaling [128,129]. Subsequently, several studies have explored the role of oxidants in various signaling cascades; however, new investigations are emerging that show kinases themselves can be regulated by oxidation. An evolutionary basis for kinase redox regulation has also been proposed [130,131]. Here, we will focus on key kinases involved in myocardial I/R injury, but please see Truong and Carroll (2013) for a comprehensive, non-I/R injury review [4].

### 4.3. PI3K/Akt Oxidation

Oxidation of PI3K has not yet been identified. However, for Akt, a redox proteome and phosphoproteome study revealed two reversibly oxidized cysteine residues (Cys60/Cys77) within Akt1 that stabilize the PIP3 binding pocket (Table 1) [132]. This finding is consistent with reports showing that H_2_O_2_ treatments leads to the release of the lipid PIP3 and activates Akt1 [133,134]. While oxidation may activate Akt1, it may have an alternate effect with Akt2. Platelet-derived growth factor is a potent activator of ROS and can inhibit Akt2 signaling through oxidation [128,135]. Akt is a key component of the RISK signaling pathway that shields the myocardium from I/R injury, yet the role of ROS-mediated Akt1 activation in I/R injury or cardioprotection is not fully understood.

Studies have indirectly implicated the oxidative activation of Akt as an important component of cardioprotection. Overexpression of thioredoxin—a major antioxidant protein—preserved Akt signaling pathways and reduces infarct size in I/R injury-induced mouse hearts [136]. This study demonstrates that possible Akt oxidation in the heart may lead to inactivation and greater I/R injury. Yet, treatment with exosomes—mesenchymal stem cell-derived factors beneficial to cardiomyocyte growth—decreased protein oxidation, such as thioredoxin, while increasing Akt phosphorylation [137]. These conflicting experiments suggest that Akt may contain unique regulatory cysteines critical for its ability to fine-tune an integrated response to stimuli. New studies examining the role of oxidized Akt in the heart are needed. Discovering the specific redox-sensitive Akt sites may inform novel interventions to shield these important residues and preserve Akt function during I/R injury.

### 4.4. AMPK Oxidation

Oxidation of AMPK is an emerging area of kinase redox modification with potential consequences in the understanding of redox regulation of metabolism. Few studies have explored the role of oxidized AMPK in the heart. In 2014, Shao et al. showed that AMPK is oxidized at murine Cys130 and 174, and both are needed to interact with Trx1 and LKB1 (Table 1) [138]. Furthermore, mutation of both cysteines destabilized endogenous AMPKα2, impaired activity, and increased infarct size [138]. This study showed that oxidation impairs AMPK. Yet, subsequent reports demonstrated that H_2_O_2_ and mitochondrial ROS stimulated AMPKα1 through the oxidation Cys299 and 304 (Table 1) [139,140]. Taken together, the impact of AMPK oxidation may be isoform- and cysteine-specific, yielding unique structural and functional consequences on cellular physiology. More studies are needed to understand if these redox modifications may influence metabolism and cardioprotection.

Polymorphisms may also provide for an “individual susceptibility” to I/R injury. Mutations in the regulatory AMPK subunits are known to cause electrophysiological abnormalities and cardiac hypertrophy [141,142,143]. Mutations (Thr400Asn) in the AMPK regulatory subunits showed an early activation of AMPK and resulted in greater infarct following I/R injury [144]. An Asn488Ile mutation, which leads to an impair conduction system, altered AMPK and rendered the enzyme insensitive to normal regulatory mechanisms, such as ATP or AMP [143,145]. Oxidant-mediated inhibition of AMPK in individuals with this polymorphism may help to reduce I/R injury by providing an alternative mechanism for regulation. More studies are needed to understand the role of oxidized AMPK in I/R injury and how it may affect I/R injury susceptibility.

### 4.5. PKA/PKG Oxidation

PKA and PKG are both known to be oxidized and form disulfide bonds to induce the activity of the kinases. The oxidation of PKA during cardiac oxidative stress was first demonstrated by Brennan et al. in adult rat cardiomyocytes following diamide treatment [146]. Subsequently, the authors found an interdisulfide bond with the RI subunit which activated PKA to induce muscle contraction [147]. This disulfide is thought to be between Cys16 and Cys37 in rats (Table 1) [148]. In 2019, Haushalter et al. showed that H2O2 treatment of adult mouse ventricular myocytes led to an increase in PKARIα activity and activation of apoptosis pathways [106]. A subsequent study demonstrated that a “redox dead”, Cys17 mutated PKARIα had larger infarcts and reduce the recovery of left ventricular function following I/R injury [149]. This cysteine mutation changed PKARIα localization to lysosomes and regulated two-pore channels and calcium transients [149]. While these indicate oxidant-activated PKA may be detrimental to the heart, in certain contexts, PKA oxidation may help to remedy arrhythmias by inhibiting potassium currents. Treating a “redox dead” PKA with H_2_O_2_ and failing to prolong action potentials or alter potassium currents has been previously reported [150]. Genetic polymorphisms in PKA can alter PDE and AKAP binding, thus altering PKA activity and cardiovascular disease susceptibility [151,152,153]. While the role of AKAP mutations in I/R injury is not fully understood, these mutation do increase the risk of myocardial infarction, arrythmias, and sudden cardiac death [154,155]. Consideration of these mutation in future studies may help to understand the oxidant-mediated regulation PKA and its influence on I/R injury. 

PKGIα is a redox sensing protein active by intermolecular disulfide bonds forms between Cys42 residues within its subunits (Table 1) [156]. These cysteines flanked by basic amino acids to promote the ionization of the thiol, allow for the formation of the PKGIα complex, and leads to vasorelaxation independent of cGMP concentrations [157]. Oxidant activated PKGIα selectively phosphorylated Ser16 in phospholamban and modulates cardiac relaxation [158]. Notably, PKGIα is also thought to be oxidized at Cys117, though this modification does not seem to impact PKG activity as much as Cys42. Bovine PKGIα mutated at sites Cys42 or Cys117 were purified and treated with H2O2, and enzyme velocity was measured (Table 1). While Cys42 mutation significantly altered enzymes velocity, Cys117 activity was not altered, yet dimers were identified [159]. The role of Cys117 is not as well characterized in the heart as Cys42. While PKGIα oxidation can lead to relaxation, oxidant activation is thought to lead to adverse cardiac outcomes. A redox dead PKGIα blunted the decrease in fractional shortening following pressure overload and prevented an excessive increase in fibrosis. Recently, Cys42 oxidation was reported to increase mTORC activity and exacerbated pressure overload-induced cardiac dysfunction [160]. These important studies have revealed that oxidant activated PKGIα blunted pressure overload-induced chamber dilation, fibrosis, and hypertrophy—hallmarks of heart failure [161]. Yet, in I/R injury cardioprotective PKGIα activation appears to occur with reduced oxidative stress [162]. Oxidant activated PKGIα in I/R injury may not occur, but studies are necessary to fully understand how PKGIα oxidation is playing out in I/R injury. 

## 5. Conclusions

Novel mechanisms of cardioprotection are needed to reduce the morbidity and mortality of ischemic heart disease. While significant progress has been made in understanding the survival pathways in the heart and their potential effect on reducing infarct, more work is crucial. By studying the kinases that influence cell survival mechanisms in the heart, the field has learned how important these proteins are, especially by controlling the activity of enzymes responsible for producing oxidative molecules. Yet, it is not fully understood how these enzymes can regulate kinases and what physiological or pathological consequences these oxidative modifications can have on kinase activity. For instance, tinkering with physiologic levels of ROS may help to condition myocardium and protect against I/R injury by modifying kinase activity prior to, or after, ischemia. Understanding these fundamental mechanisms may help to design new therapies for treating cardiovascular disease and may lead to further appreciation of the immense impact that redox molecules can have on heart physiology and pathology.

## Figures and Tables

**Table 1:** Cysteine Oxidation of Cardioprotective Kinases.

Enzyme	Cysteine (Species)	On/Off	Heart Effect	References
Akt	60, 77 (Mouse)	On	Reduces I/R injury.	[110,111,112,113,114,115]
AMPK	130, 174, 299, 304 (Mouse)	On (AMPKa1) Off (AMPKa2)	Decreases I/R injury.	[116,117,118]
PKA	16, 37 (Rat) 17 (Mouse)	On None (Mouse)	Detrimental; reduce arrhythmias.	[71,119,120,121,122,123]
PKG	42, 117 (Rat/Mouse)	On	Harmful to heart failure; protective from I/R injury.	[124,125,126,127,128,129,130]

## Abbreviations

AC	Adenylyl cyclase
AKAP	A-kinase-anchoring protein
Akt	Protein kinase B
AMP	Adenosine monophosphate
AMPK	Adenosine monophosphate kinase
ATP	Adenosine triphosphate
CaMKKb	Calmodulin-dependent protein kinase kinase
cAMP	Cyclic adenosine monophosphate
cGMP	Cyclic guanosine monophosphate
eNOS	Endothelial nitric oxide synthase
ETC	Electron transport chain
GC	Guanylyl cyclase
GKAP	G-protein-anchoring protein
GSK	Glycogen synthase kinase
H_2_O_2_	Hydrogen peroxide
I/R	Ischemia-reperfusion
LKB1	Liver kinase B1
mTORC	Mammalian target of rapamycin complex
NAPDH	Nicotinamide adenine dinucleotide phosphate
NOS	Nitric oxide synthase
NOX	NAPDH oxidase
PDE	Phosphodiesterase
pGC	Particulate guanylyl cyclase
PI3K	Phosphoinositide 3-kinase
PIP_2_	Phosphatidylinositol-(4,5) bisphosphate
PIP_3_	Phosphatidylinositol-(3,4,5) triphosphate
PKA	Protein kinase A
PKG	Protein kinase G
RISK	Reperfusion injury salvage kinase
ROS	Reactive oxygen species
SERCA	Sarcoplasmic reticulum calcium transport ATPase
sGC	Soluble guanylyl cyclase
SR	Sacroplasmic reticulum
TAK1	Transforming growth factor-b-activated kinase-1
Trx	Thioredoxin
